# A Systematic Review of the Usage of Lidocaine in Hip Replacement Surgery

**DOI:** 10.7759/cureus.37498

**Published:** 2023-04-12

**Authors:** Sarah Lu, Akshay J Reddy, Michael Fei, Himanshu Wagh, Nicholas P Iskandar, Justin Lien, Neel Nawathey, Gordon H Arakji, Rakesh Patel

**Affiliations:** 1 Medicine, California University of Science and Medicine, Colton, USA; 2 Biology, Creighton University, Omaha, USA; 3 Miscellaneous, California Northstate University, Elk Grove, USA; 4 Medicine, Campbell University School of Osteopathic Medicine, Lillington, USA; 5 Medicine, Western University of Health Sciences, Pomona, USA; 6 Health Sciences, California Northstate University, Rancho Cordova, USA; 7 Internal Medicine, East Tennessee State University Quillen College of Medicine, Johnson City, USA

**Keywords:** hip replacement, arthroplastics, osteoarthritis, lidocaine, anesthesia, general anesthesiology

## Abstract

Hip replacement procedures, professionally known as hip arthroplasty, are one of the most common orthopedic procedures. Due to the variation in this procedure, the use and types of anesthetics differ. One such commonly used anesthetic is lidocaine. Since there are currently no standardized or general procedures for the application of lidocaine for perioperative hip arthroplasty procedures, this review aims to delve into this topic. A literature review surrounding the key terms “hip replacement” and “lidocaine” was performed on PubMed. After reviewing 24 randomized control trials, statistical analyses between groups that had no lidocaine versus groups that did were performed. The results showed that there was no statistical significance between various age groups and the use of lidocaine. One percent (1%) and 2% injected into the lumbar region were the most commonly reported doses of lidocaine, with 2% often being the first test dose. Other conclusions were that lidocaine was used for general anesthesia for individuals that underwent hip arthroplasty due to an underlying condition (cauda equina syndrome, ankylosing spondylitis, etc.). Lidocaine was also used for postoperative pain relief, which is a potential concern from its addictive qualities. This investigation outlines the current stance and usage of lidocaine in perioperative hip arthroplasty while noting its limitations.

## Introduction and background

Hip replacement surgery

Hip joint damage is a common issue among the elderly population and is typically caused by the effects of aging, osteoarthritis, and other degenerative disorders. Osteoarthritis is the most common cause of hip joint damage, and it occurs when the protective cartilage that cushions the joint's bones wears down over time. As a result, bones rub against each other, causing pain, stiffness, and inflammation. Due to the increasing prevalence of osteoarthritis and other degenerative disorders, the demand for high-quality hip replacement surgery has increased significantly in the United States [1]. In fact, it is estimated that over 2.5 million people undergo hip replacement surgery every year in the country. Hip replacement surgery involves a surgical procedure known as total hip arthroplasty (THA), which aims to replace the damaged hip joint with an artificial one [1-6]. During the surgery, the head of the femur is removed and replaced with a metal ball while the hip socket is resurfaced with a plastic liner and a metal sheet. This decreases the amount of friction that occurs between the joints, which in turn lessens the amount of pain that patients feel when they move. This helps restore the hip joint's mobility and function, relieve pain, and improve the patient's quality of life. However, while hip replacement surgery is a highly effective treatment for hip joint damage, it is still a major surgical procedure that carries some risks and potential complications. Therefore, patients who are considering hip replacement surgery should consult with their doctors to weigh the risks and benefits and make an informed decision. The use of lidocaine has been proven to be effective, but there is varying literature supporting the degree of effectiveness between different surgical procedures [2]. Thus, this makes it difficult for anesthesiologists to administer lidocaine, depending on the procedure. Specifically, there is a lack of literature on the use of lidocaine in perioperative hip procedures. Hip arthroplasty can be done unilaterally or bilaterally [3-5]. Additionally, some patients do not undergo total hip arthroplasty but rather a hip hemiarthroplasty (HHP). This procedure is done for individuals that are not as active at baseline compared to participants that undergo THA. It is possible to carry out this treatment using a variety of anesthetics (lidocaine, bupivacaine, benzocaine, etc.), which can require differing injection locations [6-8]. The severity of the fracture, location, and angle is simply a few criteria that impact may treatment [7]. These factors are often not taken into consideration when using lidocaine in the literature. Depending on the case, a variety of anesthetics can be used for preoperative and postoperative pain management, as well as an anesthetic during the procedure. The objective of this study is to investigate a number of aspects pertaining to the administration of anesthesia and the patients in order to ascertain whether or not there are any aspects of the administration of anesthetics that have the potential to influence the outcomes of hip replacement surgery for patients.

## Review

Methods 

**Figure 1:**
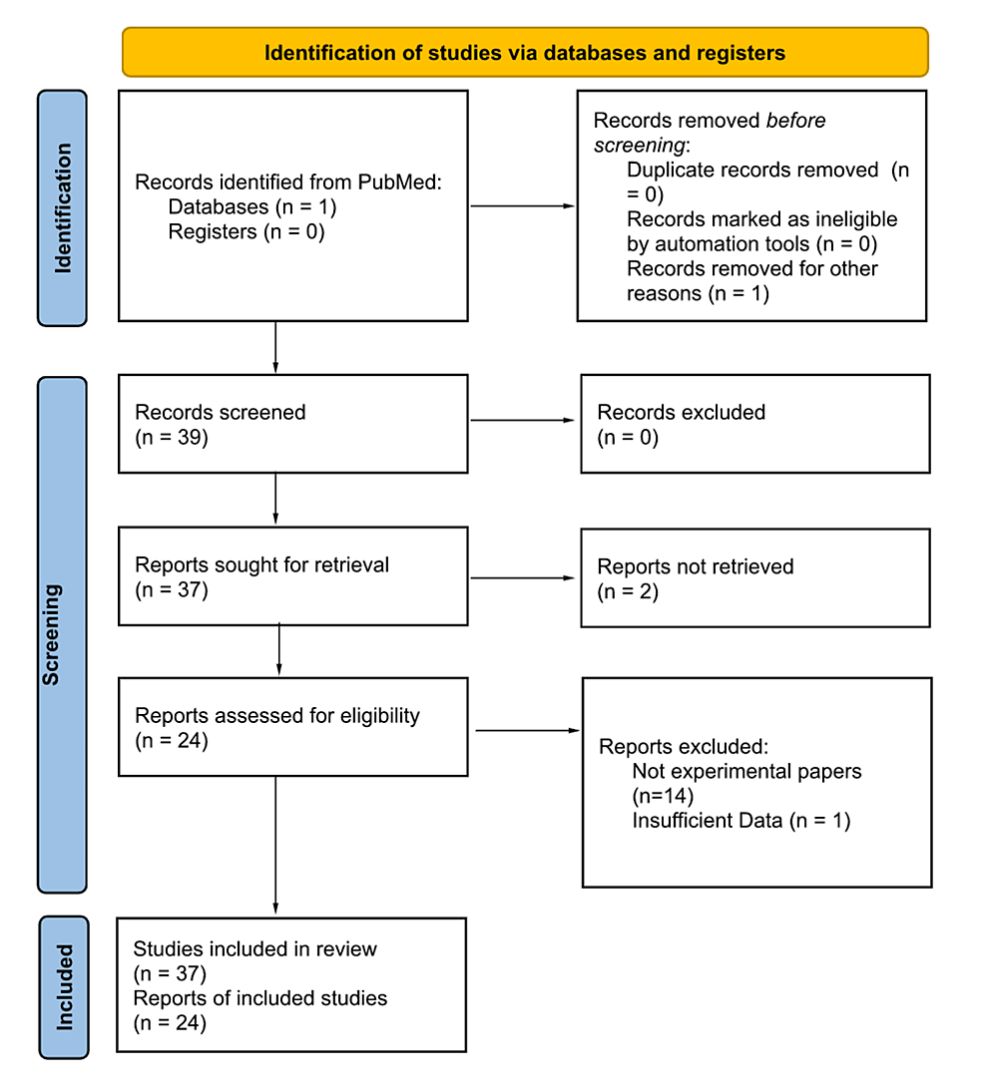
PRISMA Diagram of Studies in this Review PRISMA: Preferred Reporting Items for Systematic Reviews and Meta-Analyses

A robust search was conducted using the keywords "Hip replacement AND lidocaine." The primary search engine used was PubMed. Articles were included if they were written in English and detailed lidocaine administration in the procedure. Peer-reviewed journal articles were extracted for this paper. This initial search result yielded 39 articles. Two articles were excluded because their reports were not able to be retrieved. Articles for this review were accepted if they included the injection site, lidocaine dose, co-injection analgesia (if applicable), and route of administration. After this step, 25 papers about randomized control trials with adequately detailed methodology were included. Articles were excluded if there were no reported quantifiable or pertinent results regarding lidocaine administration, interventions, or populations. Additionally, the aforementioned criteria were extracted from each paper for analysis. A final number of 24 papers that fit the inclusion and exclusion criteria were examined for this review paper. The relatively clear exclusion criteria that were reviewed by all authors of this paper limited the risk of bias. The filtration process for this investigation can be seen in Figure 1. The studies that were analyzed for this review can be seen in Table 1 [3-27]. Statistical analysis was performed to determine if the age of the participants correlated to a higher dosage of lidocaine that was administered. First, a two-tail t-test was performed between the control group population's average age and the experimental group population's average age. Since some studies were case studies and others had large patient groups, an adjusted two-tailed t-test was performed where the sample's average age was used as an individual patient's age for that sample's population size. We also ran a two-tail t-test looking at the dose of lidocaine for patients who were under 50 years of age and patients who were over 50 years of age. Finally, with experiments that had data on both the control and experimental groups, we ran a chi-squared test. Based on the values that were calculated, there was no statistically significant data that proved that different age groups received a higher dosage of lidocaine when hip surgery was performed. This may be a point of investigation in the future, as more data is necessary to verify the results that were produced in this investigation.

**Table 1:** Summary of Lidocaine Usage in Patients With Hip Replacement Surgery *THA = total hip arthroplasty (hip replacement); **cases = how many operations done; N/A = not available

Author (year)	**Cases	Injection site	Lidocaine dose	Co-injection analgesia	Route of administration	Patient outcome	Notes	Average age
Arnunstasupakul et al., 2017 [3]	110	Posteromedial psoas	1%	Epinephrine	IM Injection	Pain rating 30 min post-injection, # of patients w/ Minimal composite score >8	Compared ultrasound-guided vs non-guided nerve block	60.3
Batra et al., 2006 [4]	1	L3-L4 space	2%	Epinephrine	Intraspinal injection	Recovery time was 24 hours	N/A	58
Berti et al., 1997 [5]	30	L3-L4 or L2-L3	2%	N/A	Intraspinal injection	Core temp	Compared heat retention w/ or w/out insulation in surgery	68.3
Casati et al., 1997 [6]	32	L3-L4 or L2-L3	2%	N/A	Control: intraspinal injection Experimental: cranial epidural catheter	Systolic bp, diastolic bp, cardiac output	N/A	58.7
Dolan et al., 2008 [8]	80	In between the pubic tubercle and the anterior superior iliac spine.	2%	N/A	Intravascular injection	Loss of sensation in the medial thigh during surgery	N/A	69.3
Frisch et al., 2018 [9]	11 w/ *THA (50 total)	Illiac spine	2%	N/A	Intraspinal	Time to discharge, length of motor blockage, time to ambulation	N/A	57.18
Gentili et al., 1998 [10]	1	L3-L4 space	2%	N/A	Intraspinal injection	Hypotension, loss of consciousness	N/A	69
Gurlit et al., 2004 [11]	66	L3-L4 space	2%	N/A	intraspinal injection	Reduction in patient pain, nausea, and vomiting	N/A	63.62
Heywang-Kobrunner et al., 2001 [12]	15	Obturator canal	1%	N/A	Intravascular injection	Significant reduction in patient pain	N/A	N/A
Kawaguchi et al., 2011 [13]	24	Epidural space	1.5%	Epinephrine	Epidural catheter insertion	Post-operative pain at mobilization was decreased	N/A	61.4
Lee & Braehler, 2017 [14]	1	Spinous process at the level of the iliac crest	1.5%	Epinephrine	Subcutaneous Injection	Hypotension	N/A	56
Lim & Kennedy, 1994 [15]	1	Femoral joint capsule	1%	Bupivacaine + ketamine	Injection	Vitals	N/A	90
Mandell 1990 [16]	5	Trochanteric bursa	2%	methylprednisolone acetate	Injection	N/A	N/A	27.6
Martin et al., 2008 [17]	58	Blood vessel	1%	N/A	IV bolus infusion	Pain detection	N/A	N/A
Modig et al., 1981 [18]	30	Oral	400 mg orally for 12 days	N/A	Oral	thrombosis	Compared tocainide vs lidocaine	64.085
Morohashi et al., 2016 [19]	1	Anterior, at Level of iliopsoas tendon	N/A	N/A	IM injection	Pain level, muscle strength	N/A	70
Nielsen et al., 2019 [20]	20	Deep to the iliopsoas muscle	5 ml in 18 mg/mL	Epinephrine	IM injection	Max force of knee extension	N/A	25
Saito et al., 2012 [21]	4	L5-S1 or hip joint	1%	N/A	Intraspinal injection	Lower leg pain	N/A	69.7
Sharrock et al., 1990 [22]	54	2 IVF above surgery	2%	epinephrine	Intraspinal	Anaesthesia felt or not	N/A	58.9
Shields et al., 2018 [23]	1	Spine	1%	N/A	Epidural catheter infusion	Cauda equina symptoms (VAS)	N/A	59
Singelyn & Gouverneur, 1999 [24]	64	L2-L3 or L3-L4	2%	Epinephrine	Intraspinal injection	Pain and satisfaction scores (VAS)	N/A	64.3
Tseng et al., 2017 [25]	1	L4-L5	2%	Epinephrine	Epidural catheter infusion	N/A	N/A	69
Wank et al., 2004 [26]	2	Anterior surface of the iliopsoas muscle at the level of the prosthetic acetabular rim	6 ml	Triamcinolone acetonide	Intraspinal needle	Pain reduction	N/A	44.5
Won et al., 2020 [27]	2	Iliopsoas tendon sheath	N/A	Steroid	IV injection	Groin pain	N/A	44.5

Lidocaine administration 

Lidocaine is a local anesthetic that is frequently used during perioperative hip arthroplasty procedures. However, there is currently no standardized procedure for its administration. During total or hemiarthroplasty hip procedures, lidocaine is commonly used as part of the general anesthesia protocol [9-11]. After the surgery, lidocaine is often administered locally as an analgesic to alleviate postoperative pain. Moreover, lidocaine has been used to manage hip arthroplasty-associated symptoms, including lumbar spinal stenosis, ankylosing spondylitis, osteoarthritis, and acute cauda equina syndrome [15-17]. Despite its widespread use, there is no agreement on the optimal dose, timing, or route of administration for lidocaine during hip arthroplasty procedures. Studies performed by Modig and Morohashi suggested that a higher dose of lidocaine may be associated with better postoperative pain control while another experiment conducted by Nielsen has found no significant difference [18-20]. Moreover, while some clinicians prefer to administer lidocaine via epidural or intravenous routes, others prefer to use local infiltration [21-23]. Additionally, there is a lack of literature on operative and postoperative lidocaine use for hip replacement procedures. The results from this review can guide physicians in using lidocaine for hip replacement patients. The studies in this review have shown that lidocaine is as good, if not a better general anesthetic during hip replacement surgeries so future studies can supplement these findings. If this is the case, there can be a standardized protocol for lidocaine administration during hip replacement surgeries. Furthermore, experiments in this study found that lidocaine injections were beneficial for postoperative pain compared to more conservative treatments such as non-steroidal anti-inflammatory drugs (NSAIDs) [8-14]. Physicians can also create standardized procedures for lidocaine use for conditions in hip arthroplasty candidates. Many studies that were reviewed stated that lidocaine was efficacious in reducing pain and improving symptoms briefly. Ultimately, lidocaine use is an effective method of reducing pain and reducing detrimental hip arthroplasty complications.

Data analysis

A chi-squared test was done on the average age of the control group versus the experimental group, which produced a value of 0.979. From this, a T-value of 0.212 was calculated. Lastly, an adjusted T-value of 0.0832 was obtained. This means that there are no significant differences between the average age at the time of surgery between the control and experimental groups. This result reduces the chances of confounding variables of lidocaine dosage given due to age differences. A T-test between doses of papers with an average age below 50 and above 50 was calculated to be 0.183, which is not statistically significant. Many hip arthroplasty operations are elective procedures, with individuals above 50 being a common population from this study. This also eliminates the biased population of hip arthroplasty is a confounding factor of lidocaine dosage. Epinephrine is observable to be a common co-injection for hip surgery, with eight studies that use epinephrine out of the 12 studies that use co-injections and a total of 44% of all studied patient cases in the review [3-27]. Epinephrine is known to both increase the intensity and prolong the actions of lidocaine. Although the exact mechanism and interaction of lidocaine and epinephrine are unclear, it has been shown that epinephrine does not increase the intraneural lidocaine, but the lidocaine content at later times is four times as high [28-33]. One of the articles suggested that this is due to the vasoconstricting effect of epinephrine, thus decreasing the blood flow in that compartment and ultimately delaying the removal of the lidocaine [33]. The use of lidocaine with epinephrine is heavily dependent on the study and the use case. It allows experiments to be done that require around 243 min of lidocaine action, which is significantly more than the duration of just lidocaine, which is around 153 mins, without the use of another analgesia like bupivacaine [34]. This was useful in studies that measured outcomes like lidocaine duration or had secondary postoperative effects [3,33]. However, there does not seem to be a correlation between when lidocaine is used with epinephrine and the type of study that is being done. Table 1 organizes all the literature used in this study with pertinent information about each study, including case number and injection site [3-27]. The most common injection site among the included studies is the L3-L4 space. The L3-L4 space is one of the possible areas of injection that falls under the category of lumbar epidural steroid injection [10-11]. Most commonly, as is the case for hip replacements, the lumbar region is effective in providing medications to the epidural space behind the spinal nerves of patients. The lumbar region consists of the lumbar vertebrae, lumbar 1 to lumbar 5. The clinicians involved in the studies examined likely chose the L3-L4 space specifically due to this region being most local and directly involved in the nerves of the hips [4-6]. The epidural space of the lumbar region is also an efficient way to provide localized anesthesia with minimal risk of contraindications that come with other injection sites. There has also been evidence that suggests epidural analgesia is advantageous in reducing the frequency of nausea and vomiting in relation to systemic analgesia. While additional studies are required to conclusively determine the most effective and safe injection site for hip replacements, the combination of factors discussed are the most probable factors in the decision for the patients to receive lidocaine through the L3-L4 space [10-11].

Dosage

The most common concentration of lidocaine used was 2%, with 11 studies reporting that concentration [3-27]. Eight studies reported using 1% lidocaine, and two studies reported using 1.5% lidocaine. The average lidocaine dose used in all papers is 1.5%. The variation in lidocaine dosage could be due to the lack of literature and protocols or physical factors. From PubMed, there were only 39 results, and there were no protocols. Thus, an explanation is that physicians lack a standardized procedure for lidocaine admission. Another consideration is that the dosages of lidocaine change based on the physical attributes of the patient. The papers used in this review did not provide adequate data on their patient populations. The lidocaine dosage could be adjusted based on each patient's BMI, comorbidities, or tolerance levels. In most studies, lidocaine was injected via intraspinal injection into the subarachnoid space of the lumbar vertebrae [3-27]. The lidocaine was used as general anesthesia in patients undergoing hip hemiarthroplasty or total hip arthroplasty. In many studies, 2% lidocaine seems to be used as a test concentration and if no subarachnoid hemorrhage symptoms were noted (nausea, vomiting, loss of consciousness, double vision, etc.). One study stated that 1-2% lidocaine produced "satisfactory surgical anesthesia" with predictable side effects [6]. Two percent (2%) lidocaine was associated with normal body temperature, normal cardiovascular responses, and quicker postoperative recovery [5,9]. Two percent (2%) lidocaine is also safe for outpatient use for postoperative anesthesia. The consistency of lidocaine doses may be due to the standardized patients undergoing perioperative hip arthroplasty procedures. Most patients are above 50 years old, as hip arthroplasty is associated with degenerative disorders of bone (ankylosing spondylosis, osteoporosis, etc.) [11-15]. The hip bears a substantial amount of weight in the body, so patients who elected for hip arthroplasty are in the healthy range for BMI (20-25) to decrease complications after surgery [17-22]. Because the patient population is relatively uniform, it is reasonable to expect the lidocaine dose to follow this trend.

Limitations

This study only included papers indexed in PubMed. This yielded a data pool of 24 papers that met the inclusion criteria, but the data set could have been bigger if papers indexed in other databases were collected. With more data, this study would have had more power, and the analysis would have been more comprehensive. The patient outcomes collected were vastly diverse, focusing on difficult-to-compare categories such as pain reduction, muscle strength changes, body temperature, vital signs, time to discharge, and complications. The low sample size made it even more difficult to compare the incidence of each of these outcomes, as there were only a few studies that focused on each of these categories. Additional sources with more comprehensive patient profiles and standardized patient outcomes could strengthen this paper. Pain reduction was the most commonly mentioned outcome, which makes it the easiest to analyze outcomes in this study. However, other outcomes, such as core temperature, were only mentioned in one paper out of the 24 that were analyzed, making it difficult to analyze trends in the data for how lidocaine influenced that particular outcome [3-27]. Most papers reported good patient outcomes after the use of lidocaine. In all papers, there was no single measure for patient outcomes. The terms for satisfactory patient outcomes were vague as well. The papers included for analysis had diverse methodologies and patient demographics. Different concurrent medications were administered in addition to lidocaine, which could have influenced the outcomes. Patients were of different ages and had different preexisting conditions such as osteoporosis or previous fracture history. Patients were also on blood thinners, diabetes medications, lipid-lowering medications, or other chronic medical treatments. Differences in variables such as these could have led to bigger differences in the patient outcome than the lidocaine dose, administration site, and method of administration. Despite the significant research that has been conducted on the administration and use of lidocaine in patients following hip replacement surgery, additional research is required to identify more optimal standards that may be used to avoid adverse effects. Currently, based on the data in this review, we are aware that the average concentration of lidocaine during hip replacement surgery is 1.5%; however, we do not have a protocol or procedure to follow if this concentration must be amended for a specific patient. Input from physicians, healthcare professionals, and researchers alike can give input for a comprehensive protocol. It may be necessary to do additional research utilizing combination medicines to address these concerns. This research may require the use of compounds such as EDTA in order to investigate the impact of various substances on the analgesic effects of lidocaine [35]. Individuals who require hip replacement surgery must have the appropriate analgesic medication in order to prevent unfavorable health effects and declining returns on treatment. This will help ease any discomfort patients may be experiencing. Possibly in the near future, AI software that analyzes X-rays will be developed to increase the number of patients diagnosed with and treated for hip fractures [36]. It is essential that further study on lidocaine's side effects be initiated to ensure the safety of hip fracture patients undergoing surgery,

## Conclusions

The purpose of this review was to provide an overview and ascertain specific conclusions concerning the usage of lidocaine in hip replacement surgery. It was determined that the most common area of application for the anesthetic was the L3-L4 space. This is most likely due to the fact that the nerves within the L3-L4 space are responsible for hip muscle motility, despite the variable hip arthroplasty procedures. According to the results, the patient outcomes of the procedures utilized were favorable, even though there was a lack of consolidated patient outcome measures across all the papers. The dosage of lidocaine and the patients’ age ranges were also cataloged in this investigation. The authors conducted statistical analysis on this data and determined that there was no statistically significant difference between the dosage of lidocaine that was utilized for patients in different age groups. Further research is necessary to validate these results and bridge the sparse literature on this topic.
